# Investigating the effects of strap tension during non-invasive ventilation mask application: a combined biomechanical and biomarker approach

**DOI:** 10.2147/MDER.S121712

**Published:** 2016-11-29

**Authors:** Peter R Worsley, George Prudden, George Gower, Dan L Bader

**Affiliations:** Southampton General Hospital, Clinical Academic Facility, Faculty of Health Sciences, University of Southampton, Southampton, UK

**Keywords:** medical device, pressure ulcers, respiratory masks, non-invasive ventilation, biomarkers

## Abstract

Non-invasive ventilation is commonly used for respiratory support. However, in some cases, mask application can cause pressure ulcers to specific features of the face, resulting in pain and reduced quality of life for the individual. This study investigated the effects of mask strap tension on the biomechanical and biomarker responses at the skin interface. Healthy participants (n = 13) were recruited and assigned two different masks in a random order, which were fitted with three strap conditions representing increments of 5 mm to increase tension. Masks were worn for 10 minutes at each tension followed by a 10-minute refractory period. Assessment at the device–skin interface included measurements of pressures at the nose and cheeks, temperature and humidity, a selection of inflammatory cytokine concentrations collected from sebum and scores of comfort. The results indicated significantly higher interface pressures at the bridge of the nose compared to the cheeks for both masks (*p* < 0.05), with nasal interface pressures significantly increasing with elevated strap tension (*p* < 0.05). One inflammatory cytokine, IL-1α, increased following mask application at the highest tension, with median increases from baselines ranging from 21 to 33%. The other cytokines revealed a less consistent trend with strap tension. The participants reported statistically greater discomfort during elevated strap tension. Temperature and humidity values under the mask were elevated from ambient conditions, although no differences were observed between mask type or strap tension. The bony prominence on the bridge of the nose represented a vulnerable area of skin during respiratory mask application. This study has shown that mask strap tension has a significant effect on the pressure exerted on the nose. This can result in discomfort and an inflammatory response at the skin surface. Further studies are required to investigate respiratory mask application for appropriate individuals with comorbidities.

## Introduction

Functional medical devices are often attached to the body with straps or tape to provide a secure fixation to the skin surface. This creates pressure and shear forces at the device–skin interface, in addition to an altered microclimate in the form of elevated temperature and humidity. These devices are typically used as interventions for critically ill individuals or those who have multiple comorbidities. Consequently, their tissues may exhibit a reduced intrinsic tolerance to withstand prolonged pressures and shear forces acting at the device–skin interface, creating a susceptibility to pressure ulcers (PUs).^1^ Indeed, PUs are defined by the National and European Pressure Ulcer Advisory Panel (2014)^2^ as “a localized injury to the skin and/or underlying tissue usually over a bony prominence, as a result of pressure, or pressure in combination with shear”. A recent study reported that 33% of all hospital-acquired PUs are related to medical devices and that those employing devices were 2.4 times more likely to have a PU of any kind.^3^ The growing awareness of medical device-related pressure ulcers (MDRPUs) has led to an international advisory panel adopting the term into their classification systems (https://www.npuap.org/).

In recent years, non-invasive ventilation (NIV) masks have been identified as potential sources of MDRPUs.^4^ NIV is delivered through a respiratory mask attached to the individual to manage a number of respiratory disorders, such as chronic obstructive pulmonary disorder and acute cardiogenic pulmonary edema.^5^ Despite the respiratory benefits of NIV being widely accepted, PU complications represent a significant burden to both the individual, in the form of pain and discomfort, as well as the health care provider. The specific incidence of NIV-related PUs has been shown to range from 5 to 50% for 2–4 hours of continuous usage and up to 100% after 48 hours of wearing a face mask.^6^

Skin damage may result from the generic mask designs employing traditional polymer materials, which may not match the shape or compliance of an individual’s facial features. In addition, there is little guidance for clinicians regarding the application of NIV masks, with straps often being applied with high tensile forces to achieve a seal with the face. Elevated tensioning of the straps has been shown to directly increase the pressure exerted on the bridge of the nose.^7^ In addition, the material at the device–skin interface influences the local microclimate.^8^ In order to create an appropriate seal, impermeable polymers are often employed the properties of which impede the normal airflow and moisture transport through the outermost skin layers.^9^ This can lead to the accumulation of moisture, creating increased friction forces at the skin interface.^10^

Research to date has focused on identifying the magnitude and location of pressure associated with the use of NIV masks for adults.^7^,^11^,^12^ Although higher interface pressures could potentially enhance the risk of damage to the skin tissues, the measure of pressure alone will not predict these areas of high risk of tissue damage.^13^ Consequently, recent research has combined physiological measures with interface pressures to establish a more robust method of monitoring tissue viability and predicting PU risk.^14^ In addition, recent evidence has shown that the inflammatory biomarkers can be used to monitor the skin response to pressure and shear, providing a potential indicator for early skin damage.^15^ This combined approach, involving both biomechanical and biomarker assessments, was adopted in the present study to examine the potential PU risk in wearing NIV masks.

## Methods

### Participants

The participants were recruited from the local university population through poster advertisement. The participants were excluded if they had the presence of facial trauma or burns, skin disease or malignancy. The protocol was approved by the ethics committee of University of Southampton, and informed written consent was obtained from the participants before testing (FOHS-ERGO-13985) and for the publication of the images.

### Test equipment

Two masks were investigated: the Philips Respironics Amara (ref no. 1090226; Respironics Inc., Murrysville, PA, USA; designated M1) and ResMed Mirage Quattro (ref no. 61226; ResMed Ltd., Bella Vista, NSW, Australia; designated M2). Both were initially fitted with reference to manufacturers’ instructions and clinical guidelines.^16^ Interface pressures were measured using a pressure monitoring system (Mk III; Talley Medical, Romsey, UK) having 18 mm diameter cells, which had a reported mean error of 12 ± 1% and a repeatability of ±0.53 mmHg.^17^ Relative humidity and temperature measurements were collected at the mask–skin interface using a Sensirion SHT75 Sensor (Stafa, Sensirion AG, Switzerland). Each sensor sampled data at 0.5 Hz and provided an accuracy in relative humidity and temperature of ±0.5% and ±0.8°C, respectively. Sebutape (CuDerm, Dallas, TX, USA) was used to collect sebum for the detection of inflammatory cytokines using a validated protocol.^18^ Subjective discomfort while wearing the mask was assessed using a 10-point visual analog scale.^19^

### Test protocol

**Figure 1:**
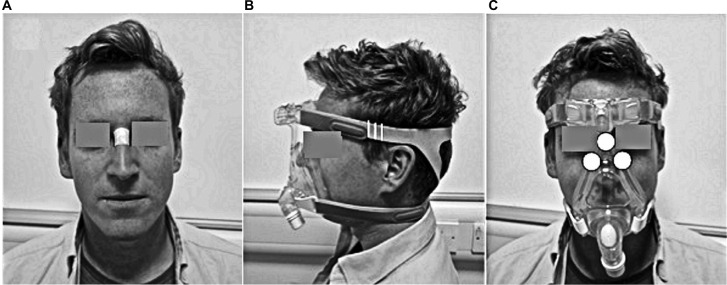
(**A**) The location of the Sebutape on the bridge of the nose pre- and post-mask applications. (**B**) Graduated marks (white vertical lines) placed on the straps of the mask to incrementally increase the strap tension. (**C**) Location of Talley pressure monitoring cells on the nose and cheeks.

All tests were performed in an environmentally controlled laboratory (temperature of 20°C, relative humidity of 50%). Prior to the application of the mask, a baseline Sebutape sample was collected for a 2-minute period. The Sebutape was attached to the bridge of the nose (Figure 1A) using blunt tweezers and gloved hands, to avoid cross-contamination of skin proteins. The participants were then fitted with NIV masks in the absence of positive pressure. On each mask, the bilateral straps on each side of the face were tensioned equally to ensure that the mask was centered on the face. Once secured, an optimum fit (T1) was defined by tensioning to a point at which two fingers could be slid between the straps and the skin.^20^ Once marked, the straps were tensioned by two further increments of 5 mm, thereby establishing tensions T2 and T3 (Figure 1B). The participants attended two sessions on non-consecutive days to test each randomly allocated mask (M1 or M2). During each of these sessions, the mask was fixed to the participant’s face using the three randomly applied tensions (T1, T2 and T3) for a 10-minute period, with the participants blinded to strap tension. For each mask and tension, pressure measurements were taken at the device–skin interface on the bridge of the nose and the superomedial aspect of left and right cheeks (Figure 1C). Three pressure values were recorded from each site following a period of 2 minutes with the mask in situ. The mean temperature and humidity measurements at the device–skin interface were then recorded for a 1-minute period. After the mask was applied for 8 minutes, the participants were asked to provide a subjective score of discomfort. Following a 10-minute period of application, the mask was removed and a second sample of Sebutape was collected from the bridge of the nose. This was subsequently followed by a 10-minute refractory period, after which the mask was reapplied with a new strap tension. All Sebutapes were coded and stored in vials at –80°C prior to biochemical analysis.

### Biochemical analysis

The Sebutape extraction process was based on the previous protocol of Perkins et al.^18^ To review briefly, the frozen tapes were thawed to room temperature and 2 mL of phosphate-buffered saline (PBS; Sigma-Aldrich Co., St Louis, MO, USA) solution was added to each vial. After immersion for 1 hour, the tapes were sonicated for 10 minutes at ±20°C, vortexed vigorously for 2 minutes and additionally mixed with a pipette tip. After refreezing overnight at –80°C, the tape extracts were thawed, vortexed for 1 minute and mixed with a pipette to recover the total extracts from the tapes. Samples from all participants (n = 13) were then processed and analyzed using immunoassay kits (Meso Scale Diagnostics, Rockville, Maryland, USA) to estimate concentrations for IL-1α. The sebum samples, from a subset of randomly allocated participants (n = 7), were separately analyzed for IL-1β, IL-2, IL-6, IL-8, IL-10 and IFN-γ (multiplex kit; Meso Scale Diagnostics).

### Data analysis

Absolute values for cytokine concentration before and after the mask intervention were obtained from the Sebutape samples, and the corresponding ratio values post- to pre-mask application were calculated for the three test conditions. Descriptive and inferential statistics were performed using IBM SPSS statistics V22 (IBM Corporation, Armonk, NY, USA). Normality was tested by the Kolmogorov–Smirnov test. Subsequently, the results were expressed as mean with standard deviation for interface pressures and relative humidity values. Non-parametric descriptors were used for cytokine concentrations, temperatures and subjective comfort. Two-way repeated measures analysis of variance and Friedman tests were used to evaluate the effect of mask type and tension. Differences were considered to be statistically significant at the 5% level (*p* < 0.05).

## Results

### Participants

Thirteen healthy participants (six males and seven females) were included in the study with a mean age of 25 years (range 21–31 years). Their mean height was 1.7 ± 0.1 m and mean weight was 73 ± 17 kg with a corresponding body mass index of 24.8 ± 3.2 kg/m^2^.

### Interface pressure

Table 1 reveals that the interface pressures at the bridge of the nose were considerably higher than the values at the cheek with median differences ranging between 34 and 77 mmHg (*p* < 0.05 for all test conditions). Although pressures on the left cheek tended to be higher than those on the right cheek for both masks, the differences were not statistically significant (*p* > 0.05). There was a significant association between strap tension and nasal interface pressures for both masks (*p* < 0.01). This association was particularly marked with the Respironics Amara (M1) mask, which revealed nasal pressures of 158 ± 54 mmHg at the highest strap tension, representing an 88% increase compared with those measured at the optimum fit (T1). The corresponding values in the interface pressures on each cheek with varying strap tensions were not statistically significantly different for either mask (*p* > 0.05).

### Cytokine analysis

**Figure 2:**
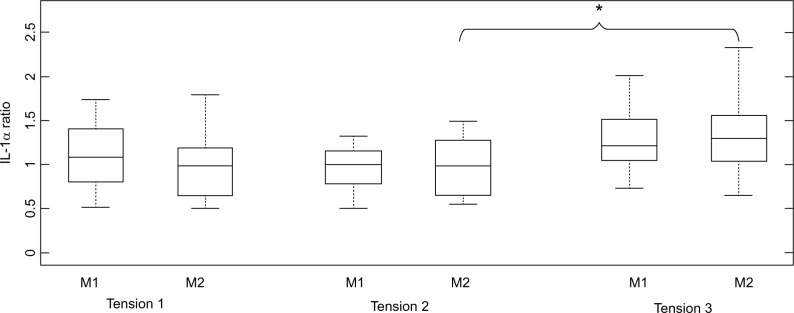
Box and whisper plots of the IL-1α cytokine ratios from pre- to post-mask application. **Note:** ^*^P-value =0.04. **Abbreviations:** M1, mask 1; M2, mask 2.

It was evident that for IL-1α, there was a general increase in cytokine ratio at the highest strap tension (T3) for both masks. Indeed, this increase in ratio was significant for M2 (*p* < 0.05), with a median ratio of 1.34 in IL-1α concentrations at T3 (Figure 2), representing increases for 10/13 (79%) of the participants. There were only modest increases in IL-1α ratio (median ratios of 0.97–1.18) between strap tension (T2) and the optimal fitting (T1), with differences not being statistically significant (*p* > 0.05).

**Figure 3:**
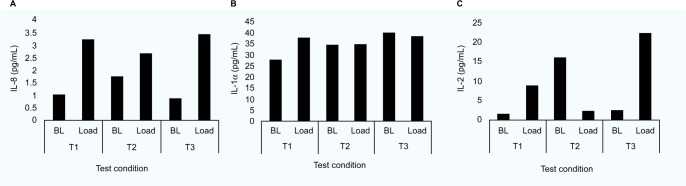
(**A**) IL-8 cytokine concentrations for participant P2 during M1 application. (**B**) IL-1α cytokine concentrations for participant P12 during M2 application. (**C**) IL-2 cytokine concentrations for participant P4 during M1 application. **Abbreviations:** M1, mask 1; M2, mask 2; T1, tension 1; T2, tension 2; T3, tension 3; BL, baseline.

Table 2 provides a summary of the pre- to post-mask ratios for the range of six cytokines in a subset of participants (n = 7). The analyses revealed more varied results, with IL-8 ratios increasing for all tensions applied to M1 (median ratios of 1.34–1.60). However, the corresponding data for M2 only revealed a major increase in the ratio at tension T2 (ratio = 2.84). No statistical significances were observed (*p* > 0.05) in any of the cytokines with respect to mask type or tension. Indeed, close examination of the data revealed a range of cytokine values with respect to strap tensions. Three distinct profiles were identified as indicated in Figure 3. For example, in some cases, cytokines were elevated post-mask application compared to baseline values for each tension (Figure 3A). Despite a statistical significance in IL-1α ratios for the highest tension in M2, some participants (e.g., P12) showed little change in concentrations pre- and post-mask applications (Figure 3B). In addition, some individuals revealed an inconsistent trend in cytokine release. This is exemplified in one case (P4, M1, IL-2) with an increase in the cytokine concentration on the application of strap tensions T1 and T3, but an apparent decrease after T2 (Figure 3C).

**Figure 4:**
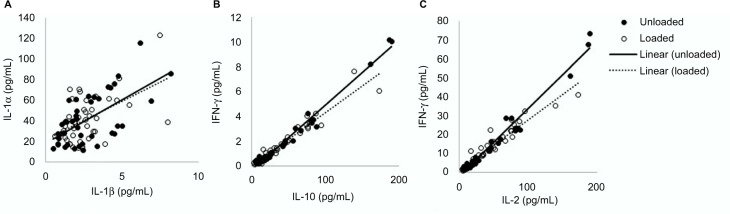
Relationship between cytokine groups: (**A**) IL-1 family, IL-1α and IL-1β; (**B**) IL type II family, IFN-γ and IL-10 and (**C**) γ family, IL-2 and IFN-γ.

Secondary analysis of the cytokine data revealed significant relationships between cytokines groups,^21^ including IL-1 family (*R* = 0.62, *p* < 0.05; Figure 4A), IL type II family (*R* = 0.93, *p* < 0.01; Figure 4B) and the γ chain family (*R* = 0.92, *p* < 0.01; Figure 4C). These relationships were evident for both loaded and unloaded samples.

### Temperature and humidity

The results revealed that the interface between the respiratory mask and the skin exhibited median values for temperature of 34°C and for relative humidity of 84% (Table 1). Analysis of these data showed that there were no significant differences between different masks or strap tensions for either skin temperature or humidity values (*p* > 0.05). These values at the device–skin interface were significantly greater than ambient conditions, for all tests, with mean ambient values of 22.3 ± 0.8°C for temperature and 56.1 ± 8.1% for humidity.

### Perceived comfort

There were statistically significant increases in subjective discomfort from optimal tension (T1) to increased strap tensions (T2 and T3) for both masks (*p* < 0.05). However, there were no significant differences in subjective comfort between masks (M1 vs. M2) for each of the corresponding strap tensions (*p* > 0.05).

## Discussion

The aim of this study was to investigate the effect of varying NIV mask design and strap tension by measuring the physical conditions at the device–skin interface (pressure and microclimate) and the reaction at the skin surface (proinflammatory biomarkers) in a cohort of healthy participants. Two commercially available masks were fitted with an optimal strap tension, and these tensions were increased by two standardized increments. The study revealed that increasing strap tension had a significant effect on interface pressures and biomarker release, in the form of elevated IL-1α cytokines. In addition, increased discomfort was reported with enhanced strap tension for both mask designs.

This study revealed that the bridge of the nose is the site exposed to the highest interface pressures. This corresponds with typical locations of respiratory mask-related PUs reported in the literature.^22^ The bony prominence on the bridge of the nose has minimal soft tissue coverage, providing limited tolerance for vulnerable skin tissues to withstand high pressures and shear forces. The effects of increased strap tension on interface pressures at the bridge of the nose have previously been reported with an associated reduction in air cushioning support at the mask–skin interface.^7^ However, the magnitude of the nasal interface pressures (range 57–75 mmHg) was markedly lower than that recorded in the present study (84–158 mmHg). These differences may have been due to the different mask designs employed and the magnitude of tension exerted by the straps on the cohort of healthy participants. The present study also reported a high degree of variability in interface pressures, with cheek values varying between individuals within each test condition (Table 1). This variability could be explained by the generic mask designs, providing limited accommodation for inter-individual differences in face and nose shapes. Indeed, it has been reported that individuals with craniofacial anomalies have a greater risk of PUs due to poor mask fit.^9^ Poor fitting masks are often over tightened to provide a seal, despite the fact that most commercially available NIV systems are designed to compensate for minor gas leakage.^23^

The present study is the first to investigate the biomarker response of the skin to NIV mask application. The concentrations of inflammatory cytokine IL-1α measured in sebum was observed to be greater following the application of the masks at the highest tension (T3). The data revealed ratio increases of 21–33% compared to unloaded skin following a refractory period. These trends were not consistently observed in other cytokines for a subset of the participants (Table 2), with varied participant-specific responses between strap conditions for individual cytokines (Figure 3). Previous studies have used sebum sampled with Sebutape to measure the inflammatory response during mechanical loading of the forearm using indenters.^24^ One such study revealed that a pressure of 100 mmHg applied for 2 hours at the volar aspect of the forearm revealed a 2.5-fold increase in IL-1α concentrations compared to the value at an adjacent unloaded control site. In a separate study, a combined biaxial load was applied at the forearm, incorporating an applied pressure of 30 mmHg and a shear force equivalent to 18 mmHg.^15^ Significant changes in IL-1α concentrations were only evident when the combined loading was applied for 30 minutes. This highlighted the temporal nature of proinflammatory cytokine release at the skin surface and may explain the variable changes in markers observed in the present study using the 10-minute loading and refractory periods between test conditions.

The results from the present study revealed relatively low correlations between IL-1α and IL-1β (*R*^2^ = 0.38). IL-1α is released from keratinocytes in response to several stimuli, acting as the primary event of inflammation. Consequently, it may have limited specificity to determine whether skin has been irritated by either mechanical or chemical insults and lacks the sensitivity to detect subtle changes to skin physiology. The analysis of IL-1α in conjunction with secondary mediators, for example IL-8, which is associated with the promotion of dendritic cell migration and recruitment of monocytes and neutrophils during the initiation phase of cutaneous inflammation,^25^ may provide more robust means to detect inflammation resulting from mechanical stimuli in the form of pressure and shear. Although the present findings from the multiple cytokine assays did not provide significant results in relation to strap tension, there is a need to establish the cytokines’ relationships with barrier disruption and impairment of healing. Indeed, previous in vivo and in vitro research has shown that multiple cytokines can simultaneously exert their effects on the same cell population, altering the function of the epidermal layers.^26^ The present study revealed highly significant correlations of cytokine concentrations within the family groups (Figure 4). Given these relationships, future studies may be able to select isolated cytokines that are indicative of multiple markers. Future studies are required, powered to examine if significant changes in secondary mediators such as IL-8, IL-6 or IFN-γ may provide more insight into the value of cytokines for the early detection of PUs.

Skin temperature and humidity data show that the interface between the skin and mask can become hot and humid, with a peak skin temperature of 36.2°C and peak skin humidity of 95.9%. These temperature values are higher than those cited for unloaded facial skin, which was reported to be 32°C around the cheek area.^27^ Indeed skin temperature in excess of 35°C has been found to have a detrimental effect on the stratum corneum by affecting its mechanical stiffness and strength,^8^ therefore increasing the risk of tissue damage.^28^ The high humidity levels at the skin interface recorded in the present study will also have a direct impact on skin health in the form of stratum corneum softening and increased cellular permeability, leading to increased susceptibility to skin irritation.^29^ Moist skin will also have an increased coefficient of friction, resulting in elevated shear forces at the device–skin interface.^30^

The present study included some methodological limitations, which include the use of young healthy participants, thus limiting the generalization of the findings. In addition, the current study did not provide positive pressure within the mask, which has been reported to produce a changing profile of interface pressures during ventilation.^11^ The mask application time was relatively short when compared to the clinical application of NIV.^31^ In addition, the recovery period of 10 minutes may not have been sufficient for the skin to fully recover to the unloaded state. A previous study has proposed a refractory period of ~20 minutes for upregulated cytokines to return to basal levels.^24^ Indeed, this was evident in some cases with some “carry over effect” of upregulated cytokines between loaded and unloaded conditions (Figure 4C). However, in clinical practice, if NIV interventions are removed for prolonged periods, patients may be at risk of oxygen desaturation. Further research is required to define exact refractory periods in which cytokine expression returns to basal levels and whether these periods are clinically viable.

### Clinical perspective

During NIV application, the clinicians’ primary focus is to attain a mask seal, as air leaks are associated with reduced comfort and efficacy of the intervention.^12^ To achieve this seal, strap tension is often increased, with the risk of pressure damage a secondary consideration.^32^ It is important to consider that the patient may not be alerted to an uncomfortable mask fit due to sedation, medication, or neurological disease or injury.^3^ Oronasal masks have traditionally been preferred for their comfort and ease of use, although other interfaces, such as full-face masks have been shown to reduce PU incidence.^33^,^34^ The development of new evidence-based devices, which protect the skin,^35^ enhanced guidance for clinical application, for example, with respect to strap tension, and the use of prophylactic interventions should also be considered for the prevention of NIV-related PUs.^36^ When device-related injury does occur, there is a need to promote accurate documentation and communication with clinical colleagues, regulatory bodies and industry. Advanced medical device surveillance systems could be considered to provide a framework for improving patient safety.^37^ Further research should monitor the interface pressures and skin physiological response of patients, who require NIV treatment over prolonged periods of application. The comorbidities of patients requiring NIV may contribute to an altered intrinsic capability for their tissues to tolerate prolonged pressure from respiratory masks.^1^ The combined measurement of physical conditions and biomarkers offers a robust objective methodology to monitor tissue damage risk in these individuals.

## Conclusion

The present study has considered both the biomechanical and biomarker responses to NIV mask application in a cohort of healthy participants. The study revealed that small increments in strap tension result in large interface pressures transferred to the bridge of the nose. This resulted in significant increases in discomfort and the release of proinflammatory cytokine, IL-1α, during the highest strap tension. It is therefore important that clinicians consider how tightly they apply the straps of respiratory masks and monitor the health of skin regularly for individuals who required NIV treatment.

## Figures and Tables

**Table 1:** Summary of outcome measure data from M1 and M2 across three strap tensions

Outcome measure	M1			M2		
	T1	T2	T3	T1	T2	T3
Interface pressure – nose (mmHg), mean ± SD	84 ± 39	132 ± 63	158 ± 54	94 ± 49	102 ± 52	121 ± 55
Interface pressure – right cheek (mmHg), mean ± SD	32 ± 18	42 ± 21	54 ± 24	39 ± 12	41 ± 21	36 ± 20
Interface pressure – left cheek (mmHg), mean ± SD	37 ± 17	30 ± 13	42 ± 24	32 ± 21	38 ± 26	33 ± 29
Temperature (°C), median (range)	34 (32–35)	34 (33–36)	34 (32–36)	33 (29–35)	34 (32–35)	34 (32–35)
Humidity (% RH), median (range)	83 (67–91)	84 (75–92)	84 (77–92)	84 (77–96)	80 (67–93)	84 (73–94)
Discomfort score (VAS/10),* median (range)	4 (0–7)	5 (0–7)	6 (0–7)	4 (0–7)	5 (0–7)	5 (1–8)

**Note:**

* 1 = very comfortable, 10 = very uncomfortable.

**Abbreviations:** M1, mask 1; M2, mask 2; T1, tension 1; T2, tension 2; T3, tension 3; RH, relative humidity; VAS, visual analog scale.

**Table 2:** Summary of ratio changes in the cytokines pre- to post-mask application

Pre- to post-mask ratio	M1			M2		
	T1	T2	T3	T1	T2	T3
IL-1β, median (range)	0.75 (0.62–2.14)	1.18 (0.56–2.44)	1.27 (0.50–2.14)	0.73 (0.34–1.88)	1.44 (0.74–3.08)	0.70 (0.63–1.30)
IL-8, median (range)	1.34 (0.39–3.20)	1.45 (0.18–2.75)	1.60 (0.43–5.28)	0.53 (0.26–2.17)	2.84 (0.27–7.11)	0.62 (0.34–1.46)
IL-2, median (range)	2.60 (0.56–6.13)	0.90 (0.11–2.60)	0.53 (0.19–9.49)	0.34 (0.18–1.77)	2.00 (0.24–21.18)	0.50 (0.31–4.26)
IL-6, median (range)	1.44 (0.20–2.05)	0.71 (0.21–2.55)	1.01 (0.55–6.10)	0.90 (0.45–2.36)	1.30 (0.62–2.99)	0.80 (0.58–2.35)
IL-10, median (range)	2.24 (0.41–4.60)	1.03 (0.14–2.18)	0.73 (0.15–9.14)	0.37 (0.26–1.95)	2.49 (0.26–23.50)	0.66 (0.34–3.45)
IFN-γ, median (range)	2.01 (0.38–3.16)	0.87 (0.19–1.77)	0.66 (0.26–5.85)	1.32 (0.29–2.05)	1.17 (0.35–10.00)	0.62 (0.39–2.40)

**Abbreviations:** M1, mask 1; M2, mask 2; T1, tension 1; T2, tension 2; T3, tension 3.
